# Red Kale (*Brassica oleracea* L. ssp. *acephala* L. var. *sabellica*) Induces Apoptosis in Human Colorectal Cancer Cells In Vitro

**DOI:** 10.3390/molecules28196938

**Published:** 2023-10-05

**Authors:** Kamila Rachwał, Iwona Niedźwiedź, Adam Waśko, Tomasz Laskowski, Paweł Szczeblewski, Wirginia Kukula-Koch, Magdalena Polak-Berecka

**Affiliations:** 1 Department of Microbiology, Biotechnology and Human Nutrition, University of Life Sciences in Lublin, 8 Skromna Street, 20-704 Lublin, Poland; iwona.niedzwiedz@up.lublin.pl (I.N.); adam.wasko@up.lublin.pl (A.W.); magdalena.polak-berecka@up.lublin.pl (M.P.-B.); 2 Department of Pharmaceutical Technology and Biochemistry and BioTechMed Center, Faculty of Chemistry, Gdańsk University of Technology, Gabriela Narutowicza Str. 11/12, 80-233 Gdańsk, Poland; tomasz.laskowski@pg.edu.pl (T.L.); pawel.szczeblewski@pg.edu.pl (P.S.); 3 Department of Pharmacognosy with Medicinal Plants Garden, Medical University of Lublin, 1 Chodzki Str., 20-093 Lublin, Poland; virginia.kukula@gmail.com

**Keywords:** plant bioactive compounds, anti-cancer action of plant compounds, cytotoxicity of natural compounds against cancer cells

## Abstract

This article presents the results of studies investigating the effect of red kale (*Brassica oleracea* L. ssp. *acephala* L. var. *sabellica*) extract on cancer cells (HT-29). The cytotoxicity of the red kale extract was assessed using MTT and LDH assays, while qRT-PCR was employed to analyze the expression of genes associated with the p53 signaling pathway to elucidate the effect of the extract on cancer cells. Furthermore, HPLC-ESI-QTOF-MS/MS was applied to identify bioactive compounds present in red kale. The obtained results indicated that red kale extract reduced the viability and suppressed the proliferation of HT-29 cells (the IC_50_ value of 60.8 µg/mL). Additionally, mRNA expression analysis revealed significant upregulation of several genes, i.e., *casp9*, *mapk10*, *mapk11*, *fas*, *kat2 b*, and *ubd*, suggesting the induction of cell apoptosis through the caspase-dependent pathway. Interestingly, the study revealed a decrease in the expression of genes including *cdk2* and *cdk4* encoding cell cycle-related proteins, which may lead to cell cycle arrest. Furthermore, the study identified certain bioactive compounds, such as sinigrin, spirostanol, hesperetin and usambarensine, which could potentially contribute to the apoptotic effect of red kale extracts. However, further investigations are necessary to elucidate the specific role of these individual compounds in the anti-cancer process.

## 1. Introduction

Colon cancer, also known as colorectal cancer (CRC), is the third most prevalent type of cancer worldwide. In 2020, approximately 2 million new cases of CRC were diagnosed and nearly 1 million deaths reported. New cases of CRC are reported mainly in older people of both genders, but recent years have also seen an increase in incidence among younger adults. In addition, an increased risk of colorectal cancer is linked to genetic determinants but also to unhealthy lifestyle habits, including a diet rich in highly processed foods, smoking, excessive alcohol consumption, and lack of physical activity. These lifestyle factors, among others, can accelerate the production of free radicals. The adverse effects of free radicals on cells, particularly resulting in DNA damage, can contribute to the development of cancer. Crucial to the successful treatment of colorectal cancer is its early detection and subsequent selection of appropriate therapy. Treatment selection depends on the location of the tumor, the stadium of the disease, as well as the patient’s overall condition. Common treatments include chemotherapy, radiation therapy or removal of cancerous tissue. However, colorectal cancer remains a major health challenge, and the development of innovative therapies is essential to reduce the disease burden and improve patient outcomes [1].

Bioactive compounds of plant origin are becoming increasingly important in cancer prevention and therapy. Plant compounds acting as natural antioxidants help protect the body against the harmful effects of free radicals [2]. The damage to important structures of human cells (proteins, lipids, nucleic acids, carbohydrates) induced by the action of free radicals is considered one of the main causes of civilization diseases, such as cardiovascular diseases, cancer, diabetes and neurodegenerative diseases. In recent years vegetables from the genus *Brassica* receive scientific attention, as numerous studies have proved that a diet rich in cruciferous vegetables is associated with lowering the risk of cancer and other chronic diseases [3,4].

Red kale (*Brassica oleracea* var. *sabellic*a) is a member of the *Brassicaceae* family, which plays an important role in the cuisine and diet of many people in Europe, the United States, and Asia [5]. A high intake of these vegetables may bring a lot of health benefits linked to their bioactive compound such us vitamin C, polyphenols and glucosinolates (GLS) [6]. Within the bioactive compounds present in plants of the *Brassicaceae* family, GLS, exhibit notable anti-inflammatory and antioxidant capabilities in the body. Indeed, many studies indicate that the degradation products of glucosinolates, such as indoles, nitriles, isothiocyanates or nitriles, are characterized by high antitumor activity and may be an interesting alternative to conventional cancer therapeutic strategies [7]. The anticancer effects of these compounds may include suppressing the transcription of cancer repressor genes, inhibiting the growth of cancer cells by blocking the cell cycle or inhibiting nuclear translocation and enzyme activity [8]. In addition, flavonoids, saponins and some alkaloids present in these plants can exert anti-inflammatory, antioxidant and antitumour activity. First attempts have already been made to identify the effect of kale on cancer cell proliferation, however, despite promising results, there is still a scarcity of comprehensive studies to link individual bioactive compounds contained within this plant to a specific biological effect. These studies also do not account for the effect that is exerted when this plant is subjected to simulated digestion in vitro. Moreover, there is a lack of studies aimed at understanding the mechanism of action of the substances contained in this plant at the cellular level by analyzing the effect of the extract from this plant on the expression of genes related to the process of tumorigenesis.

In this regard, the aim of this study was to determine the potential anticancer activity of the in vitro digested red curly kale extract against human colorectal cancer cells (HT-29) by assessing cell viability and proliferation. Furthermore, to understand the mechanism of action of the compounds contained in the kale extract, its effect on the expression of genes related to the p53 pathway was examined. In addition, using HPLC-MS fingerprinting, we attempted to identify compounds that could potentially be responsible for the pro-apoptotic effect of the tested extract.

## 2. Results

### 2.1. Red Kale Extract Decrease Viability and Proliferation of HT-29 Colon Cancer Cells

**Figure 1:**
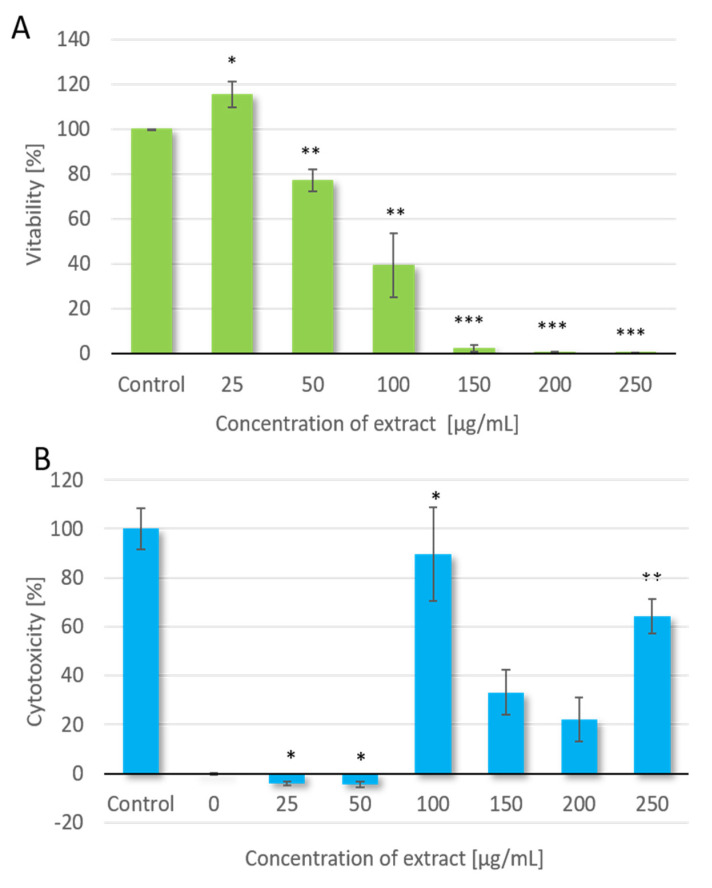
Viability [%] of HT-29 cells after treatment with red kale extract assessed using MTT assay (**A**) and cytotoxicity [%] of extract assessed by LDH assay (**B**) * *p* value < 0.05; ** *p* < 0.005; *** *p* < 0.0001.

The effects of various concentrations of red kale extract on HT-29 cells were evaluated by MTT and LDH assays after 72-h exposure. The results were presented as % of viable cells in reference to the control (cells untreated with the extract tested). The results revealed that the viability of HT-29 cells decreased in a concentration-dependent manner. Red kale extract at the lowest concentration tested (25 μg/mL) positively affected cell viability and probably stimulated cell divisions (Figure 1A). At higher concentrations of the extract, a reduction in cell viability was observed, which increased with the concentration of the extract. The growth inhibitory effect (IC_50_) value was obtained at a concentration of 60.8 μg/mL.

LDH assay was used to assess the level of plasma membrane damage in HT-29 line cells treated with red kale extract. In this assay, it was observed that the extract at a concentration of 25 μg/mL caused no change in the cells compared to the control, and the highest cytotoxicity was reported for the extract at a concentration of 100 μg/mL.

Determination of LDH activity in cells treated with kale extract showed a different cytotoxicity than that shown by MTT assay (Figure 1B). The differences are attributable to the different operating principle of the two methods. MTT is based on the number of viable cells, while the intensity of the color generated in the LDH assay correlates directly with the number of lysed cells. It is suggested that the action of the extract at concentrations greater than 100 μg/mL results in decreased cell proliferation, which directly affects the number of cells. This explains the lower cytotoxicity at higher extract concentrations due to fewer cells that may have been lysed rather than the lower cytotoxicity of the extract at such concentrations.

### 2.2. mRNA Expression Levels of Cell Apoptosis-Related Genes was Increased in HT-29 Cells Treated with Red Kale Extract

To further investigate the mechanism involved in cytotoxicity induced by red kale extract, we examined the mRNA expression of 92 target p53 signaling associated genes in control samples and those treated with red kale extract in concentration of 100 μg/mL.

From among the four candidate genes for endogenous control (*18S, GAPDH, HPRT1, GUSB*) we decided to use *GAPDH* as reference gene. *GAPDH* mRNA was found to be the most suitable internal reference for this qPCR assay since it was the least variable under experimental treatments (its C_T_ values had minimal standard deviations between samples).

**Figure 2:**
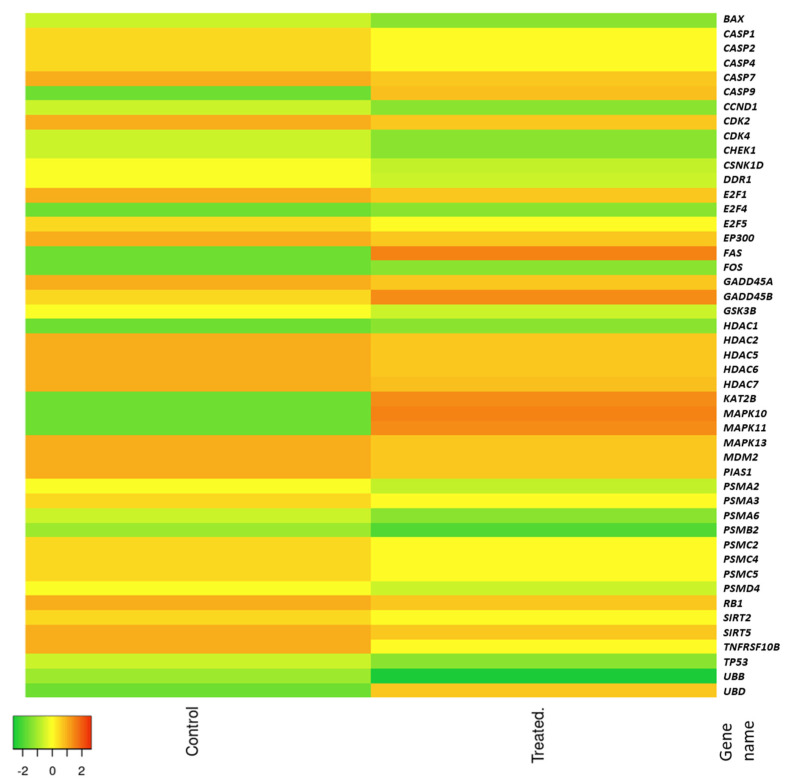
Heat map showing the fold changes in gene expression between the control and red kale extract-treated human colon carcinoma cell line HT-29; genes that demonstrate differential fold change patterns in red kale-treated cells are summarized.

Results obtained with the application of qRT-PCR technique revealed that the expression levels of *casp9, fas, fos, kat2b, mapk10, mapk11, ubd, hdac1* and *gadd45a* mRNA significantly increased following the treatment of HT-29 cells with kale extract in concentration of 100 μg/mL for 48 h in comparison with control, indicating caspase-dependent apoptotic activity of the kale extract. The most significant difference was observed for *casp9*, *fas, kat2b, mapk10, mapk11* and *ubd.* On the other hand, the most noticeable decrease in expression levels of genes such as *tp53*, *bax, ccnd1, ubb,* genes encoding Ser/Thr protein kinases (*cdk2*, *4* and *chek1*), histone deacetylases (*hdac 2, 5, 6, 7*), proteasome 20 S and 26 S subunits (*psma 2 and 3, psmb2, psmc2, 4 and 5, psmd4*), sirtuin (*sirt2* and *5*), ubiquitin ligase (*mdm2* proto-oncogene) and ubiquitin *ubb* was shown (Figure 2).

### 2.3. HPLC-MS Fingerprinting of the Extracts

The applied chromatographic techniques provided the separation of the constituents of kale juice. The fingerprints of all tested samples were alike, and they did not show many signals in the mass chromatograms.

So far, the scientific literature lacks data on a tentative identification of metabolites present in plant extracts subjected to the digestion process. The influence of enzymes on the composition of extracts and on the final structure of plant derived secondary metabolites was significant in this case so it was impossible to find many matching metabolites in mass spectrometry databases.

The study on the qualitative composition led to the identification of several components that are presented in Table 1. The identification was performed on the total ion chromatogram (see Figure S1 in the Supplementary Materials) based on the high-resolution mass measurement data, the MS/MS spectra of the compounds that were compared with the scientific literature and with the mass databases, like Metlin. In the tested sample, sinigrin belonging to the glucosinolates was tentatively identified. In the extracts the presence of citric, malic and pyroglutamic acids was confirmed together with hesperetin, propylglutaric acid and unknown spirostanol type saponin. Also, a large peak of usambarensine was recognized in the recorded spectra. This compound was previously found in *Brassica napus* extracts [9].

## 3. Discussion

Colorectal cancer is one of the most common malignancies in both sexes, with a high mortality rate. Due to their properties, plant-derived bioactive compounds have great potential for use in cancer prevention and therapy as an alternative to the cytostatics currently in use.

In the present study, we demonstrated that kale reduced the viability and inhibited the proliferation of colon cancer cell of HT-29 line. The effect is dose-dependent with IC_50_ value of 60.8 µg/mL. Previously, other plant extracts were shown to have similar effects on the viability and proliferation of HT-29 cells, indicating that phytochemicals have potential anticancer activity. Laka et al., revealed a cytotoxic effect of *Drimia calcarata* bulb extracts against the HT-29 cells. A decrease in cell viability was shown in MTT test, and the IC_50_ values obtained occurred at a concentration of 125 μg/mL [10]. Similarly, MTT assay demonstrated that *Brucea javanica* fruit extract has an antiproliferative effect on HT-29 cells, with a IC_50_ value of 25 ± 3.1 µg/mL after 72 h of treatment [11]. Extracts obtained from *Calotropis gigantea* (flowers, leaves, roots, and root bark) were also found to exhibit cytotoxicity in in vitro models. MTT assay showed a dose-dependent decrease in viability of the HCT116 and HT-29 cells treated for 24 h with *C. gigantea* extracts. The obtained IC_50_ values were in the range of 44 to 86.7 μg/mL [12]. Purified extracts from white and red pomace, and grape seeds were also reported to reduce proliferation and viability of HT-29 cells. LDH test revealed cytotoxic effect of crude grape extracts in concentration of 250 mg/mL after 24 h of incubation [13]. Moreover, *Cynara cardunculus L. subsp. scolymus* (L.) (artichoke) extract in concentration of 1 mg/mL decreased viability of HT-29 cells in a concentration- and time-dependent manner [14].

Analysis of changes in mRNA expression levels of selected p53 pathway genes induced in HT-29 cells treated with red kale explain the observed cytotoxic effect of the red kale extract on the HT-29 cells. Genes that were up-regulated by treatment with red kale extract are mainly engaged in cell death by apoptosis and anti-cancer effects. Casp9 plays a central role in apoptosis and is a tumor suppressor, Fas is a death receptor involved in cascade of caspases that mediates apoptosis, while *ubd* encodes ubiquitin D which targets proteins for proteasome degradation and can mediate apoptosis in a caspase-dependent manner. Kat2 B plays a direct role in transcriptional regulation similarly to Hdac1, which is histone deacetylase engaged in regulation of gene expression. Mapk10 and 11 are Mitogen-Activated Protein Kinases, involved, among others, in programmed cell death. Analyzing genes with increased expression following treatment of cells with red kale extract, the observed effect is most likely a consequence of apoptosis induction in a caspase-dependent pathway.

Regarding the genes that were down-regulated in cells treated with red kale extract, these include *bax* (BCL2 associated X protein, apoptosis regulator) which encodes a protein that acts as anti- or pro-apoptotic regulator that can be involved in a wide variety of cellular activities. The expression of *bax* gene is regulated by the tumor suppressor p53. This protein is encoded by the *tp53* (Tumor Protein p53) gene and was shown to be down-regulated in cells treated with red kale extract. The p53 protein responds to diverse cellular stresses to regulate expression of target genes and mutations in this gene are universal across various cancer types. The p53 protein was shown to transcriptionally regulate *mdm2.* Overexpression of this gene was previously detected in different types of cancers [15]. In our research, we observed higher levels of Mdm2 mRNA in samples treated with red kale extract than in control HT-29 cells. Mdm2 protein was found to promote tumor formation through targeting p53 and other suppressor proteins for proteasomal degradation. Also, the expression level of sirtuin, which may play a role in tumor initiation, promotion, and progression, was reduced in HT-29 cells after treatment with red kale extract [16]. Additionally, *psma3* and *psma6* encoding proteasome 20 S subunits, whose expression was decreased by kale extract, was previously shown to be upregulated in some cancer types [17]. Similarly, *ccnd1* gene encoding cyclin D has been shown to be misregulated in many cancer types. Cyclins consist of a group of proteins which function as regulators of CDK kinases. Cyclin D is a regulatory subunit of CDK4, whose activity is required for cell cycle G1/S transition. Decrease in levels of the mRNA expression of *cdk2* and *cdk4* encoding two of the serine/threonine kinases was also observed in cells treated with red kale extract. Proteins encoded by *cdk2* participates in cell cycle regulation and regulates progression through the cell cycle. Product of *cdk4 i*s crucial for cell cycle G1 phase progression. Mutations in this gene as well as in D-type cyclins related to Cdk4 was shown to be associated with tumorigenesis of a variety of cancers. Decreased expression of *ccnd1* encoding cyclin D and *cdk4* may be responsible for alteration in cell cycle progression and may induce a G_0_/G_1_ cell cycle arrest. These results indicate that red kale extract affects the down-regulation of several genes previously identified as overexpressed in cancer cells and also genes encoding cell cycle-related proteins. Decreased expression of aforementioned genes in cancer cells treated with red kale extract may indicate its anti-tumor properties and suggest that reduced proliferation of cancer cells may be the result of changes in the cell cycle or even cell cycle arrest.

Similarly, it was observed that the antitumor activity of the Baneh extract against human breast cancer is partly caused by cell cycle arrest and downregulation of *ccnd1* and *cdk4* expression [18]. Reduced expression of *cdk4* inducing a chemopreventive effect was also observed by Shah et al. in their study on the impact of flaxseed extract rich in secoisolariciresinol diglucoside on colorectal cancer [19]. In turn, studies on the anticancer potential of violacein against breast cancer cells also showed that this compound increased expression of *fas, casp9, bax, p53* and decreased expression level of *mdm2*. Thus, violacein induced apoptosis of cancer cells through TNF-α and p53 dependent mitochondrial pathways. [20]. *Brucea javanica* ethanolic extract has also been demonstrated to exhibit anticancer activity and induce apoptosis in HT-29 colorectal cancer cells through activation of extrinsic and intrinsic pathways [11].

The findings from this study demonstrated the high anticancer potential of the compounds contained in red kale. HPLC-ESI-QTOF-MS/MS fingerprinting of curly kale extracts was used to identify compounds that could be responsible for the observed biological effect exerted by the extract. Among the compounds identified in red kale subjected to prior in vitro digestion, sinigrin and spirostanol type saponins, usambaresine and hesperitin are known for their biological activity. Sinigrin is a compound that belongs to the glucosinolates, present in various plants of the *Brassicaceae* family, with well-documented anticancer activity [15,21,22,23,24,25,26]. Studies have shown its effect on changes in gene expression in cancer cells. As a result of down-regulating *bcl-2*, *mdm2* and up-regulating *bax* and *p53* expression, sinigrin stimulated the release of cytochrome c. According to experimental evidence provided by changes in gene expression following sinigrin administration, this compound is capable of causing cell cycle arrest by apoptotic events and inhibiting cancer growth through a p53-dependent mechanism [15]. Moreover, by preventing the activation of the NLRP3 inflammasome or the NF-B/MAPK pathways in macrophages, sinigrin reduces the generation of inflammatory mediators [23]. Sinigrin also induce overexpression of caspase-3 in DU-145 s cells and increase apoptosis [24].

Saponins are a group of amphipathic glycosides that are found in a wide range of plant species. They can be employed in many different therapeutic compositions and are crucial to maintaining human health. Saponins are essential in the inhibition of numerous molecular pathways, including PI3 K/AKT, AKT/MAPK, EGFR/PI3 K/AKT, PI3 K/AKT/mTOR, and RNF6/AKT/mTOR. These compounds have been shown to exert cytotoxic effects on the cancer cell lines such as HepG2, Hela, MDA-MB-231, MCF-7, NCI-H460, HT1080, H1299, A549, SGC7901, and LN229 [27]. Saponins usually exhibit anti-tumorigenic effects through a variety of anticancer mechanisms due to the significant structural heterogeneity of their structures [28]. Steroidal saponins are well known for their low toxicity and great efficacy in the prevention and treatment of cancer. In vitro and in vivo studies have shown that steroid saponin compounds have revealed that steroid saponin compounds have a variety of anticancer properties, including the ability to prevent tumor invasion and metastasis, induce apoptosis and autophagy, and reduce proliferation [29]. Spirostanol saponin derivative, named RCE-4, has shown growth inhibitory and apoptosis-inducing effects on human cancer cells (CaSki, HT-29 and CNE2 cell lines). RCE-4 altered the Bax/Bcl-2 ratio by upregulating *bax* expression while downregulating *bcl-2* expression [30].

In the examined material, usambaresine was also identified, which belongs to a large group of plant secondary metabolites called alkaloids. This compound is a colorant and a metabolite proved to exhibit antioxidant properties. Alkaloids are a diverse group of organic compounds characterized by the presence of one or more nitrogen atoms in their structures. Of the 27,000 diverse alkaloids that have been identified, approximately 17,000 have exhibited a range of pharmacological properties, including anticancer activities [11]. One of the alkaloids, Isostrychopentamine (ISP) was shown to exert the anticancer activity on HCT-116 and HCT-15 human colon cancer cells with IC_50_ values of 7.0 µM for HCT-116 and 15.0 µM for HCT-15. Additionally, ISP was found to arrest the cell cycle in the G2-M phase and activate various pathways leading to apoptosis [31]. Another study demonstrated that usambaresine exhibit cytotoxic effects on cancer cells. It also revealed that treatment with this compound is linked to a loss of G1 phase cells and a significant rise in the sub-G1 region, which is indicative of apoptotic cells. During the G1 phase, cells prepare for entry into the cell cycle, and duplicate their DNA in the S phase. Moreover, it causes DNA fragmentation and increased proteolytic activity of DEVD-caspases [32].

Analysis of red kale also revealed the presence of a flavonoid representative—hesperitin (HSP). Flavonoids, which are divided into flavonols, flavonones, flavones, isoflavones, flavanols, and anthocyanins, are the largest group belonging to polyphenols. This diverse group of compounds, present in fruits, vegetables, grains, bark, roots, stems, flowers, tea, and wine, is well known for its broad health-promoting activity [33]. HSP is a flavonoid that exhibits various biological properties. Investigations of the structure-activity relationship model of HSP allowed the determination of its potential biological activities, which include anticancer or chemopreventive activity. Chemopreventive properties are mainly related to antioxidant, anti-inflammatory and radical scavenging activity. The compound acts at different stages of tumor development, inhibiting tumor growth by directing multiple protein targets of cells simultaneously. Among others, it affects caspases, Bcl-2 and Bax for induction of apoptosis, and COX-2, MMP-2 and MMP-9 for inhibition of angiogenesis and metastasis [34].

## 4. Materials and Methods

### 4.1. Plant Material

The red curly kale leaves used in this study were harvested from our own cultivation (located in southeastern Poland) in October 2020. The fresh curly kale leaves were washed, dried, and freeze-dried by lyophilization and then stored in a freezer at −20 °C until analysis.

### 4.2. Preparation of Red Curly Kale Leaf Extract and In Vitro Digestion

The sample was prepared by mixing 1 g of freeze-dried red curly kale leaves with 15 mL of 20 mM phosphate buffer (pH 7.0) and 24-h incubation of samples with agitation. Then, the cells were disrupted by sonication (Sonics VCX 750 Vibra-Cell™, ultrasonic processor, Newtown, CT, USA) in ice (6 × 30 s, 5 min breaks on ice, 50%). Insoluble particles were removed by centrifugation (10,000× *g* rpm, 20 min, 4 °C), and the supernatant was transferred to a new tube. In vitro gastrointestinal digestion of aqueous extracts was performed as described in our previous work [35]. In brief, the first stage of digestion was carried out with amylase (oral digestion phase), followed by pepsin at pH 1.7 (gastric phase). Subsequently, the pH was raised to 6.5 and pepsin activity was inhibited (small intestine phase), then bile salts and pancreatin were added and incubation continued at pH 6.5 [35]. Samples after digestion were centrifuged (10,000× *g* for 30 min at 4 °C) and then frozen (−80 °C for 24 h). Subsequently, the freeze-drying process was carried out (Labconco, Kansas City, MO, USA).

### 4.3. Cell Culture

The human Caucasian colon adenocarcinoma cell culture were obtained from American Type Culture Collection (ATCC, Manassas, VA, USA). Cell culture of HT-29 cells was performed as previously described [36]. The cells were routinely maintained at 37 °C and 5% CO_2_ in RPMI 1640 (Sigma, Poole, UK) containing 10% fetal bovine serum (FBS), 2 mM L-Glutamine, penicillin (100 U/mL) and streptomycin (100 mg/mL).

### 4.4. MTT Cytotoxicity Assay

Antiproliferative potential of red kale extract in terms of anticancer assessment was evaluated for the cell viability against HT-29 cell line. The cytotoxicity of red kale extract was determined with 3-(4,5-dimethylthiazol-2-yl)-2,5-diphenyltetrazolium bromide (MTT). For MTT assay HT-29 cells were seeded in 96-well microplates at a concentration of 5 × 10^4^ cells per well, in 100 μL culture medium (RPMI with 10% FBS). Cell cultures were incubated for 24 h at 37 °C and 5% CO_2_ After the incubation period, medium was discarded from cell cultures and fresh medium (RPMI with 2% FBS) containing tested red kale extracts were added. Cells were exposed to kale extract at concentrations of 25, 50, 100, 150, 200 and 250 μg/mL (in triplicates). The control comprised samples without addition of the test substance, while the background control was the medium alone without cells or extract. Cells were incubated with the extracts for 72 h at 37 °C and 5% CO_2_ Subsequently, 10 µL of freshly prepared MTT solution (5 mg/mL in PBS) was introduced into each well. After a 3 h of incubation (37 °C, 5% CO_2_), 100 µL of solubilization solution (10% SDS in 0.01 N HCl) was added to each well in order to stop the reaction and dissolve the formed formazan crystals. Samples were incubated for 24 h (37 °C, 5% CO_2_) and absorbance at λ = 570 nm and 680 nm was determined spectrophotometrically using a Microplate Reader (EL800 Universal Microplate Reader; Bio-Tek Instruments; Winooski, VT, USA). The amount of formazan formed was directly proportional to the number of metabolically active cells.

### 4.5. Lactate Dehydrogenase (LDH) Assay

LDH assay was conducted according to the manufacturer’s protocol (LDH Cytotoxicity Assay Kit II, Sigma-Aldrich, St. Louis, MO, USA). Briefly, cells were collected, washed with fresh RPMI medium, and seeded in 96-well plate at a concentration of 5 × 10^4^ cells per well in 100 μL culture medium (RPMI with 10% FBS). Cells were incubated in an incubator (37 °C, 5% CO_2_) with curly kale extracts in final concentrations of 25, 50, 100, 150, 200 and 250 μg/mL, in triplicates. Plate was gently shaken and centrifuged at 600× *g* for 10 min to pellet the cells. A total of 10 μL of the clear medium solution from each well was transferred into an optically clear 96-well plate. 100 μL of LDH Reaction Mix was added to each well, mixed and incubated for 30 min at room temperature. A Microplate Reader was used to measure the absorbance of the samples at a wavelength of 450 nm. Cytotoxicity measurements using LDH assay were performed after 0, 24, 48, 72 and 96 h of incubation with curly kale extracts. Determinations of each sample were performed in triplicate. Simultaneously, measurements were performed for control samples: a background control that contained culture medium alone without cells, a low control with cells not treated with the investigated extracts, and a high control (positive control) where 10 μL of Lysis Solution was added to the cells. Cytotoxicity (%) was calculated as: [(A450 test sample) − (A450 low control)/(A450 high control) − (A450 low control)] × 100.

### 4.6. RNA Isolation and Genomic DNA Removal from RNA Preparations

RNA was isolated from HT-29 line cells cultured in RPMI medium with 10% FBS and in the same medium supplemented with red kale extract at a final concentration of 100 ng/μL. After harvesting by trypsinization, cells were washed with a PBS buffer and frozen at −70 °C. For RNA isolation, an RNA purification kit (ThermoScientific GeneJet RNA purification kit) was used and the procedure was performed according to the manufacturer’s recommendations. To an aliquot of 5 × 10^6^ cells, 600 μL of Lysis buffer containing β-mercaptoethanol was added. Samples were briefly vortexed for 10 s and 360 μL of ethanol was added. Lysate was transferred to an RNA purification column and centrifuged (12,000× *g*). The column was washed using Wash Buffers and subsequently RNA from the column was eluted using nuclease-free water. Genomic DNA was removed from samples using DNAase (A&A Biotechnology; Gdynia; Poland). A total of 1 U DNAase per 1 μg of RNA was used. The reaction was performed at 37 °C for 30 min and stopped by the addition of EDTA at final concentration 5 mM. DNAase was then inactivated by incubation at 75 for 10 min. Quality and quantity of obtained RNA was assessed using the Nanodrop2000 Spectrophotometer. The RNA was then used to synthesize cDNA.

### 4.7. cDNA Synthesis and Quantitative Real-Time PCR

The RNA was transcribed to cDNA using 1 μg of template RNA and the cDNA synthesis kit, according to the manufacturer’s guidelines (ThermoScientific Revert Aid First Strand cDNA Synthesis Kit). cDNA synthesis was conducted in 42 °C for 1 h and reaction was terminated by heating samples at 70 °C for 5 min. The reverse transcription reaction product was directly used in the qPCR reaction.

Comparative mRNA expression analysis was performed using real-time quantitative PCR (StepOnePlus™ Real-Time PCR System, Thermo Fisher). TaqMan Fast Advanced Master Mix and TaqMan Gene Expression Array Plate were used (TaqMan™ Array Human p53 Signaling 96-well Plate). The assay allows for simultaneously analysis of the expression of 92 genes associated with p53 signaling and 4 candidate genes for endogenous control. The following PCR conditions were applied: 50 °C for 2 min, 95 °C for 1 min and 40 cycles of denaturing at 95 °C for 1 s and annealing/extension at 60 °C for 20 s. Amplification of specific products was verified by dissociation curve analysis. The data obtained from the assay were analyzed using relative quantification (ΔΔCt algorithm). As an internal control, the GAPDH gene was selected. Statistical significance between treated groups and controls was determined by two tailed Student’s t test, and *p* < 0.05 was considered significant.

### 4.8. High-Performance Liquid Chromatography Coupled to Electrospray Ionization Quadropole Time-of-Flight Tandem Mass Spectrometry (HPLC-ESI-QTOF-MS/MS) Fingerprinting of Red Kale Extracts

A platform composed of an HPLC chromatograph (HP1200 Series, Agilent Technologies, Santa Clara, CA, USA) equipped in a degasser, a binary pump, an autosampler, and a PDA detector—with a QTOF-MS/MS mass detector (6500 Series, Agilent Technologies, Santa Clara, CA, USA) was used for the fingerprinting and purity control of curly kale samples. The chromatographic analysis was performed in an optimized method using a Zorbax Eclipse Plus RP-18 chromatographic column (150 mm × 2.1 mm, dp = 3.5 µm, Agilent Technologies, Santa Clara, CA, USA) and the following gradient of acetonitrile with 0.1% formic acid (solvent B) in 0.1% formic acid (solvent A) was used: 0 min—1% of B, 20 min—40% of B, 45 min—95% B, 48 min—95% of B, 48.5 min—1% of B. The run lasted 55 min. The flow rate was set as 0.2 mL/min, the temperature of the thermostat at 20 °C, UV detection as 210, 254, 280, 365 and 320 nm and the injection volume was 10 µL.

The mass spectrometer was operated in negative ion mode in the mass range of 100–1700 u to study the composition of phenolic constituents, with the temperature settings of 275 and 325 °C for gas and sheath gas, respectively, gas flows of 12 L/min, nebulizer pressure of 35 psig, fragmentor voltage of 110 V, capillary voltage of 3000 V and skimmer voltage of 65 V. Qualitative Navigator (version B.10.00) and Agilent Mass Hunter Data Acquisition (version 10.1) programs produced by Agilent Technologies (Santa Clara, CA, USA) were used to acquire the spectra and process the recorded data.

### 4.9. Statistical Analysis

The results of the experiments were collated in MS Excel 2013 (Microsoft Corporation, Redmont, WA, USA). Statistical data were analyzed with a one-way ANOVA test and post-hoc Dunnett’s test (all columns compared to the control) using STATISTICA 13.3 (StatSoft, Cracow, Poland). This study used p-values to describe statistically significant data; * *p* < 0.01, ** *p* < 0.005, *** *p* < 0.001 were considered significant compared to control.

## 5. Conclusions

In the present study, the effect of red kale extract on the mRNA expression of 92 genes related to the p53 signaling pathway in HT-29 cells was investigated for the first time. In addition, an effort was made to identify compounds potentially responsible for the anticancer effects of the tested extract. Our results suggest that treatment with red kale may have significant effects on the regulation of genes responsible for cellular processes such as apoptosis and cell cycle control in HT-29 cells. Further research is needed to gain a comprehensive understanding of how the various bioactive compounds present in red kale contribute to the observed changes in gene expression and their potential implications in colorectal cancer. This knowledge would enable the isolation of the most effective anti-cancer compounds from red kale which could consequently lead to their potential use in colorectal cancer therapy.

## Figures and Tables

**Table 1:** Qualitative analysis of curly kale samples—HPLC-MS fingerprint and identification data.

No	Rt (min)	m/z Calculated	m/z Experimental	Error (mmu)	RDB	MS/MS Fragments	Proposed Compound/Suggested Formula
1	4.359	358.0272	358.0266	1.66	3	-	Sinigrin C_10_ H_17_ NO_9_ S_2_
2	30.6	431.2241	431.2200	9.53	18	409, 171, 152	Usambarensine C_29_ H_28_ N_4_
3	12.8	301.0718	301.0733	5.09	10	-	Hesperetin C_16_ H_14_ O_6_
4	4.192	191.0181	191.0181	0.47	3	129, 111, 87	Citric acid C_6_ H_8_ O_7_
5	2.52	133.0142	133.0142	0.35	2	115, 71	Malic acid C_4_ H_6_ O_5_
6.	4.28	128.0353	128.0359	−4.52	3	82	Pyroglutamic acid C_5_ H_7_ NO_3_
7.	10.7	173.0819	173.0816	1.91	2	-	Propylglutaric acid C_8_ H_14_ O_4_
8.	18.33	622.3359	622.3385	−4.23	8.5	554	A spirostanol type saponin C_33_ H_51_ O_1_1

Rt—retention time, error—error of m/z measurement, RDB—rings and double bonds number.

## Data Availability

Data presented in this study are available upon request from the corresponding author.

## Supplementary Materials

The following supporting information can be downloaded at: https://www.mdpi.com/article/10.3390/molecules28196938/s1, Figure S1: The HPLC-ESI-QTOF-MS/MS mass chromatogram of the analysed extract, recorded in the negative ionization mode that was used for the compositional analysis of the sample.
