# Supplementary material for: Red Kale (Brassica oleracea L. ssp. acephala L. var. sabellica) Induces Apoptosis in Human Colorectal Cancer Cells In Vitro
Source: Molecules. 2023 Oct 5;28(19):6938. doi: 10.3390/molecules28196938 (PMC10574217; doi:10.3390/molecules28196938)
Supplement: Supplementary file 1 [file molecules-28-06938-s001.zip › molecules-2598941-supplementary.pdf]

SUPPLEMENTARY FILE

Red Kale (*Brassica oleracea* L. ssp. *acephala* L. var. *sabellica*) Induces Apoptosis in Human Colorectal Cancer Cells In Vitro

Kamila Rachwał [1,*], Iwona Niedźwiedź [1], Adam Waśko [1], Tomasz Laskowski [2], Paweł Szczeblewski [2], Wirginia Kukuła-Koch [3], Magdalena Polak-Berecka[1]

Figure S1. The HPLC-ESI-QTOF-MS/MS mass chromatogram of the analysed extract, recorded in the negative ionization mode that was used for the compositional analysis of the sample

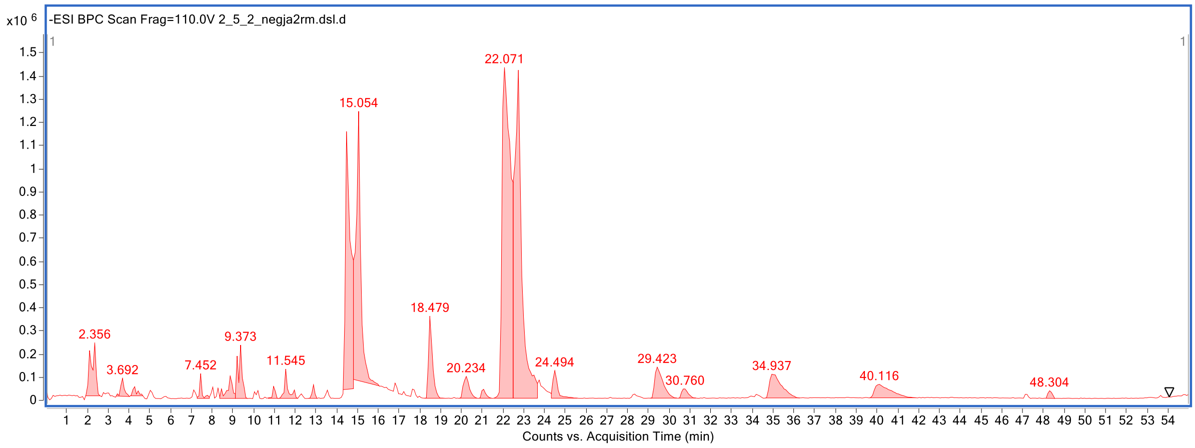

The chromatogram presented above shows the fingerprint of the extract that is obtained after digestion. That is why the alignment of peaks is not characteristic for the plant itself. The major peaks visible at ca. 15th and ca. 22nd minutes come from the bile acids that were added to the sample during the digestion process. That is why the identity of these peaks was not mentioned in the table 1 in the body of the manuscript.
